# Loneliness in Older Migrants: Exploring the Role of Cultural Differences in Their Loneliness Experience

**DOI:** 10.3390/ijerph20042785

**Published:** 2023-02-04

**Authors:** Honghui Pan, Pamela Qualter, Manuela Barreto, Hannelore Stegen, Sarah Dury

**Affiliations:** 1 Faculty of Psychology and Educational Sciences, Adult Educational Sciences, Vrije Universiteit Brussel (VUB), 1050 Brussels, Belgium; 2 Brussels Interdisciplinary Research Centre on Migration and Minorities (BIRMM), Vrije Universiteit Brussel (VUB), 1050 Brussels, Belgium; 3 Society and Ageing Research Lab, Vrije Universiteit Brussel (VUB), Pleinlaan 2, 1050 Brussels, Belgium; 4 Manchester Institute of Education, University of Manchester, Manchester M13 9PL, UK; 5 Department of Psychology, University of Exeter, Exeter EX4 4QG, UK; 6 Research Foundation Flanders (FWO), 1000 Brussels, Belgium

**Keywords:** loneliness, culture, older people, older migrants, social environment, social relationship, coping, linear regression, cultural aspects, social capital, ageism, coping strategies

## Abstract

Introduction and background: The scientific literature suggests the necessity of studying loneliness from a broader social perspective. This article aims to broaden the research on loneliness in older migrants by exploring the role of cultural differences through the lens of the social environment (as measured in social capital, discrimination, and ageism) and social situation (as measured in relational mobility, childness, and marital status). Based on Hofstede’s Individualism Index, older migrants involved in the BBC Loneliness Experiment (N = 2164) were classified into three groups: cultural migrants (i.e., from a collectivist to individualist culture) (N = 239), migrants with a similar culture (i.e., within an individualist culture) (N = 841), and ageing non-migrants (N = 1084). Objectives: The two main objectives were (1) to compare the levels of loneliness among these three groups, and (2) to unravel how different influencing factors, such as the social environment, social situation, coping strategies, and personal characteristics, are related to loneliness. Methods: Bivariate analyses were performed to determine the differences in the loneliness, social environment, social situation, and personal characteristic variables between the groups, with adjusted p-values according to the Bonferroni correction to limit the potential for type I errors (α = 0.005). Multiple linear regressions were performed to unravel the relationships between loneliness and the different influencing factors, namely the social environment, social situation, coping strategies, and personal characteristics. Results: The bivariate analyses show no significant difference in loneliness across the three groups. The multiple linear regressions demonstrate that the social environment (i.e., social capital, discrimination, and ageism) is significantly associated with loneliness. Social capital acts as a protective factor for cultural migrants (β = −0.27, *p* < 0.005, 95% CI [−0.48, −0.05]), similar-culture migrants (β = −0.13, *p* < 0.005, 95% CI [−0.25, −0.03]), and non-migrants (β = −0.21, *p* < 0.001, 95% CI [−0.28, −0.12]). Discrimination and ageism are both risk factors for loneliness across the three groups. Social situation, as measured in married/cohabitation status and relational mobility, shows a significant association with loneliness in the non-migrants and similar-culture migrants but not the cultural migrants. In terms of individual resources for coping strategies, engagement in active coping is protective for all three groups. Non-coping, the unawareness of any coping strategies, is a risk factor, while passive coping shows no significant association. Discussion: The results show that the structural factor of the social environment in which older migrants’ find themselves, rather than their culture of origin, is more important for older migrants’ feelings of loneliness in later life. A favorable social environment with high social capital and low levels of discrimination and ageism protects against loneliness in the ageing population across cultures. Practical implications for loneliness interventions for older migrants are put forward.

## 1. Introduction

Loneliness is an emerging public health concern [1], with older adults being at elevated risk of loneliness due to old-age-related vulnerabilities such as living alone, loss of family, and chronical disease [2]. Loneliness can be defined as an “unpleasant experience that occurs when a person’s network of social relations is deficient in some important way, either quantitatively or qualitatively” [3], and it is characterized by negative emotions, such as sadness and anger [4]. Older migrants are a sub-group of older people who might have different experiences of loneliness from ageing locals, since migration often influences the trajectories of social connections with family, friends, and communities [5]. The ethnic cultural background of older migrants is important in determining their participation and belonging patterns in the host society, which have important implications for their loneliness [6]. However, there are few comparative studies that distinguish between the experiences of migrants who are more or less culturally different from the ageing local population.

In the current study, we explore how culture is related to experiences of loneliness among older migrants by focusing on three groups of ageing adults: cultural migrants, similar-culture migrants, and ageing locals. The categorization of these three groups of older migrants is based on Hofstede’s Individualism Index [7]. The main research objectives are to (1) compare these three groups in terms of their loneliness levels, and (2) to unravel how different influencing factors, such as the social environment, social relationships, coping strategies, and personal characteristics, are associated with loneliness across the three groups.

## 2. Theoretical Framework and Hypotheses

### 2.1. Loneliness in the Ageing Population: The Different Characteristics of Cultural Migrants, Similar-Culture Migrants, and Non-Migrants

Migrants who relocated across cultures (e.g., from China to the UK) could have different cultural norms than those held in the host society, especially when it comes to how people relate to each other. The dimension of collectivism-individualism has been one of the most widely used constructs in cross-cultural studies to explain differences in cultures [8]. A critical difference in this cultural dimension is the tightness or closeness of people’s social relations [9]. According to Hofstede [9], in an individualist society, the “ties between individuals are loose; everyone is expected to look after him- or herself and his or her immediately family only”, while in a collectivist society, people “from birth onwards are integrated into strong, cohesive in-groups, which throughout people’s lifetime continue to protect them in exchange for unquestioningly loyalty” [9] (pp. 148–175). The cultural implication of this for loneliness is that older migrants coming from a collectivist culture into an individualist host society can have higher expectations of social connectedness and greater belonging needs than the new society can meet, leading to a discrepancy between the desired and actual social contact and, therefore, to loneliness. Migrants can also find certain social behaviors considered normal in their culture to be less accepted in their new cultural environment [10]. This can cause rejection and unsatisfying social relationships, thereby exacerbating feelings of loneliness.

Migrants can come from very different cultural backgrounds that might be rather distant from the host society. Adaptation, the process of change through which individuals become better suited to their environments [11], can often be more difficult for cultural migrants than for those who migrate within similar cultures (e.g., from one individualist society to another). For example, the cultural shock experienced by older South Asian migrants in Canada contributes to their experiences of loneliness [12]. De Jong Gierveld and colleagues [6] revealed that older migrants in Canada who are more familiar with cultural elements in the host society, for instance, those who have a similar culture and language, appear not to experience higher feelings of loneliness than non-migrants, whereas those who originate from more culturally distant countries do. A recent study in Belgium shows that compared with non-migrants, migrants have different levels of loneliness depending on their origin [13]. Compared with non-migrants in Belgium, migrants coming from Southern Europe have the highest level of loneliness, whereas migrants from Northern Europe do not differ from non-migrants in terms of their loneliness levels [13]. Accordingly, we put forward our first hypothesis:

**Hypothesis** **1** **(H1).** *Older migrants who migrate from a more distant culture have higher levels of loneliness than migrants from a similar cultural background*.

### 2.2. Loneliness in Older Migrants: The Influencing Factor of (Changed) Social Environment

For older migrants, relocation to another country is a significant life event [14]. In the process of establishing a new life in a foreign land, a mix of unfavorable factors can put older migrants at increased risk of loneliness. These factors include lower socioeconomic status [15], acculturative stress [16], loss of social support [17], and language barriers [12]. Fortunately, older migrants can draw on their ethnic culture to be resilient [18] (p. 133) and combat negative feelings of loneliness [19]. Older migrants, when compared with non-migrants, often have higher levels of life satisfaction, which has been referred to as the “happiness paradox” [20].

Older migrants might experience loneliness differently from those without a migration background for a number of reasons. The first is the changing of social networks, as migration often entails leaving behind an already established social network in their home country [21]. Another factor is the cultural distance or difference from the culture of the host country, which could lead to difficulties in socializing with others, eventually leading to higher levels of loneliness in older migrants who do not share a similar language and culture [6]. Lastly, discrimination can exclude older migrants from meaningful social networks and sources of social support, ultimately reducing their social capital [22]. However, those who experience discrimination often turn to their (ethnic) community for social support, and that can act as a protective factor against high levels of stress [23]. This is similar to the model of rejection and identification in the case of ethnic minorities proposed by Branscombe and colleagues [24]. In sum, there are reasons to expect that migrants might experience more loneliness than non-migrants, although there is also a rationale to expect resilience among migrants. Therefore, how these elements of the social environment (e.g., discrimination) relate to loneliness in older migrants needs more academic attention. Accordingly, we put forward the second hypotheses:

**Hypothesis** **2** **(H2).** *Different elements of the social environment (e.g., discrimination) are associated with loneliness differently for different groups of older migrants*.

### 2.3. The Influencing Factors of the Social Situation for the Ageing Population

The factors that influence loneliness include social participation, socioeconomic status, health, social contact, and belonging patterns [25,26]. These factors might carry varied weights for ageing migrants from different cultural backgrounds. Perhaps the best researched is the emphasis on close or familial relationships in collectivist cultures. Older collectivist migrants often place more emphasis on family relationships or relationships with their adult children [21]. Filial piety received from adult children protects older Chinese migrants in the US from loneliness [27], while not having adult co-residing children is a risk factor for ageing migrants’ loneliness [28]. In this sense, being childless in older age might weigh more heavily for collectivists than individualists. Research also shows that adults in individualist societies tend to have higher levels of relational mobility and engage more actively in close relationships than those in collectivist societies [29]. Accordingly, we put forward the following hypothesis:

**Hypothesis** **3** **(H3).** *Different elements of the social situation (e.g., childness and relational mobility) are associated with loneliness differently for different groups of older migrants*.

### 2.4. Coping with Loneliness from a Cultural Perspective

Ageing migrants can use different coping strategies to deal with the unpleasant feelings that characterize loneliness. In a favorable social environment (e.g., the availability of social support), social capital can offer resources and trust to help older migrants cope with loneliness [10]. Thus, older migrants might adopt coping strategies that include active or problem-focused coping and passive or emotion-focused coping [30]. Active coping with loneliness aims at solving the problem by increasing the amount of one’s social contact, while passive coping relates to the cognitive reappraisal (e.g., acceptance and positive reframing) of unfavorable situations and seeking ways to avoid directly dealing with loneliness, which can include engaging in distracting activities in order not to think about loneliness.

The coping strategies associated with loneliness in older migrants are seldom examined from a cultural perspective. Collectivist and individualist cultural orientations are likely to influence older migrants in terms of how they cope with an undesired situation such as loneliness. Taylor and colleagues [31] showed that (collectivist) Asians in the U.S. are less likely to utilize social support than (individualist) Europeans when developing coping strategies. Collectivist individuals may be more concerned about their relationships with others (e.g., to avoid embarrassment and protect group harmony). Regarding the choice of loneliness coping styles, older Asian adults are more likely to adopt passive coping due to their “perception of ageing as a natural process and social isolation and/or loneliness is part and parcel of life; here they accepted their situations and try to seek joy in solitude” [17]. Accordingly, we put forward the third hypothesis:

**Hypothesis** **4** **(H4).** *Different coping strategies are associated with loneliness differently for different groups of older migrants*.

By offering answers to the above hypotheses, the current study aims to address the following two research questions through focusing on three groups of older people (cultural migrants, similar-culture migrants, and non-migrants):

1. Do cultural migrants experience higher levels of loneliness when compared with similar-culture migrants and non-migrants?
2. How are different influencing factors, such as the social environment, social situation, different coping strategies, and personal characteristics, associated with loneliness for different groups of people?

## 3. Methods

### 3.1. Data Collection

The data used in this study are from the BBC Loneliness Experiment, which was launched on BBC radio channels (i.e., BBC Radio 4 and BBC World Service) and other media channels such as newspapers and television to recruit participants. The survey was developed by Claudia Hammond (British Broadcasting Corporation, BBC), Prof. Christina Victor (Brunel University London), Prof. Manuela Barreto (University of Exeter), and Prof. Pamela Qualter (University of Manchester), and it was funded by the Wellcome Trust. The data collection lasted three months from February to May 2018. The participants took an average of 45 min to complete the online survey. The questions of the survey used in this study can be found in the Appendix A. Participants were notified of the research objectives and their right to withdraw from the research without giving any reason.

### 3.2. Participants

A total of 54,988 people completed the survey in a voluntary manner, of whom 14,884 people were aged 55 years old and above and resided in the United Kingdom of Britain and Northern Island. The research population of this study consists of older people aged 55 and over. It was difficult to define a minimum age or cut-off age for older people, as there exist different perceptions about age, such as chronological age, social age, and biological age [32]. According to the World Health Organization (WHO), the minimum age is 60 years [33]. There are other gerontological studies defining people aged 55 and over as young older people for example [34]. We choose the cut-off age of 55 because for older people with a migration background, health problems do occur at an earlier age due to accumulated disadvantages, and they feel older earlier than their peers [35].

Among the group of 14,884 older adults, 1096 UK residents aged 55 years and above had a migration background from 73 countries. Based on their country of origin, the respondents were given a score according to Hofstede’s’ Individualism Index, which is a continuous 0–100 measurement [7]. The index ranges from 6 (Guatemala) to 91 (the US), and a higher score means a higher level of individualism. Using a cut-off point of 48, the respondents were assigned to either a collectivist or individualist origin. In our sample, the older migrants come from 45 collectivist countries (e.g., China, Iran, Kenya) and 28 individualist countries (e.g., the US, the Netherlands, New Zealand).

In the BBC sample, we have 13,788 older people without a migration background, 841 with a migration background from an individual culture, and 239 with a migration background from a collectivist culture. To avoid comparing hugely uneven sample sizes (13,788 vs. 841 vs. 239) and to harmonize the samples, this study used a matched case control design [36] to select a comparable sample of older people born in the UK (N = 1096) based on marital status (i.e., single, cohabitating, in a relationship, married, separated, divorced, and widowed) and self-perceived socioeconomic status. The case-control matching was conducted using SPSS software to reduce the selection bias and improve the internal validity [36]. We based the choice of matching variables (i.e., socioeconomic status and marital status) on the theoretical framework in the existing literature. For instance, Van Tilburg and Fokkema [25], in their comparative study of loneliness across three groups of people (i.e., non-migrants, migrants with Turkish origin, migrants with Moroccan origin), made similar choices in selecting a comparable sample of non-migrants to match the much smaller sample size of migrants. After the selection of the matched sample of non-migrants, we implemented a cross-sectional study to explore the association between loneliness and possible predictive variables across these three groups (1084 non-migrants, 239 cultural migrants, and 841 similar-culture migrants).

## 4. Measurements

**Dependent variable**. Loneliness was measured using the four-item UCLA loneliness scale [37]. The participants were asked to indicate the frequency of their feelings of lack of companionship, being left out, isolation, and feeling in tune with people around them based on a Likert scale ranging from one (never) to five (always). The UCLA scale was reliable, with a Cronbach’s alpha of 0.84.

**Independent variables**. The social environment, social situation, coping strategies, and personal characteristics were used as independent variables for predicting loneliness.

First, the **social environment** includes social capital, discrimination, and ageism. Social capital was measured using a seven-item scale [38] with items including “People in this neighborhood are willing to help their neighbors” and “This is a close-knit or tight neighborhood where people generally know one another”. The responses ranged from one (strongly disagree) to five (strongly agree) (α = 0.82). Discrimination was measured using the short version of the everyday discrimination scale, which includes five statements [39]. One of the five statements was “You are treated with less courtesy or respect than other people”. The participants were asked to rate their experience of discrimination on an ascending scale from one (never) to seven (almost every day) (α = 0.77). Subsequently, they were asked to indicate the main reason for the experience of discrimination: ancestry or national origins, gender, race, age, religion, height, weight, physical attractiveness, physical disability, mental health, sexual orientation, education or income, or other, and they could choose multiple reasons. For this study, we simply averaged across all the items to obtain a score of the extent to which the participants experienced discrimination across all of those identities. Ageism was measured by asking the participants to what extent they agreed or disagreed with the statement that old-age was a time of loneliness using a scale from one (strongly disagree) to five (strongly agree).

Second, the **social situation** was measured in terms of marital status (1 = married [including those in civil partnerships] or cohabiting, 2 = divorced [including formerly in a civil partnership, which is now legally dissolved] or separated [but still legally married], 3 = widowed [including surviving partner from a civil partnership], 4 = single [never married or never in a civil partnership], 5 = in a relationship [and not living together]), having child/children or not (1 = yes, 0 = no), and relational mobility. Relational mobility was measured based on the participants’ responses to twelve statements [29]. They were asked to think about their immediate community and to indicate to what extent they agreed with each item by using a 1–6 scale (1 = strongly disagree, 6 = strongly agree, α = 0.90). An example item is “Even if these people were not completely satisfied with the group they belonged to, they would usually stay with it anyway”.

Third, the **coping strategies** for loneliness were measured by asking the respondents about their self-perceived solutions that were effective at reducing feelings of loneliness. More precisely, they were asked “Please think about which of these possible solutions to loneliness you, or others you know, have found to be effective at reducing feelings of loneliness. Please select all that apply.” The respondents were given 20 possible solutions to choose from: (1) joining a club; (2) finding activities that distract you when you are on your own; (3) dedicating time to work, study, or hobbies; (4) looking for a new job; (5) setting out to introduce yourself to all your neighbors; (6) moving to a new area; (7) re-engaging with your church, mosque, or equivalent; (8) telling someone else that you’re feeling lonely; (9) talking to friends and family about your feelings; (10) deciding to invite people to be friends without fearing rejection; (11) setting out to look for the good in every person you meet; (12) deliberately starting a conversation with anyone you interact with, e.g., in shops; (13) seek counselling; (14) finding new non-social activities and pastimes; (15) finding new friends; (16) using the internet for support; (17) carrying on as normal and waiting for the feeling to pass; (18) giving yourself time to think about why you’re feeling lonely; (19) finding new social activities and pastimes; and (20) changing your thinking to be more positive. The respondents were also given the choice of “I do not know any solutions”. The solutions numbered 1, 4–7, 10, 12, 15, 16, and 19 were labeled as active coping strategies because they relate to efforts that are aimed at initiating new social contacts. The remaining 10 strategies were labeled as passive coping because they are about finding activities that temporarily distract people from loneliness. In the analysis, the variable of active coping was computed using the sum of the active solutions, while the variable of passive coping was computed using the sum of the passive solutions. Non-coping was measured by the response to the choice of “I do not know” (1 = not being aware of any solutions, 0 = being aware of at least one solution).

Fourth, demographic variables were also collected. Age, gender, years of education received, employment status, income, and self-perceived health were included as personal characteristic variables. The respondents were asked to indicate their age in years and choose their gender from four choices (male, female, other, prefer not to say). Regarding gender, those who answered “other” or “prefer not to say” were excluded due to the small sample size and the potential ambiguity of such responses. Gender was coded (1 = male, 0 = female). Education was measured in the number of years of education that they had completed from primary school to college or post-graduate study. The respondents were also asked to indicate their employment status (full-time in work, part-time in work, full-time student, part-time student, non-paid work, and unemployed). Employment status was coded (1 = full-time work or part-time work, 0 = full-time or part-time student, non-paid work or unemployed). Income was measured by asking how they feel their needs are met by the financial resources that they have (i.e., money), and they could answer either very well, fairly well, or poorly. Income was coded (1 = fairly well or very well, 0 = poorly). Self-perceived health was measured by asking how they would rate their current health status if 10 represents the healthiest that they could be and 0 represents the poorest health.

### Analytical Strategy

To answer the first research question concerning the possible variance in loneliness and the influencing factors among these three groups, statistical analyses of one-way ANOVA and Chi-square tests were used to determine the differences in loneliness, social environment variables, social situation variables, coping strategies, and personal characteristics. When conducting the bivariate analysis, we included the z-test with adjusted p-values according to the Bonferroni correction to limit the potential for type I errors (α = 0.005). To answer the other research question concerning the associations between loneliness and its influencing factors, multiple linear regressions were performed to unravel the associations between the different influencing factors and loneliness among the three groups of non-migrants, cultural migrants, and similar-culture migrants.

## 5. Results

### 5.1. Sample Characteristics

Table 1 presents the characteristics of the three groups: non-migrants (N = 1084), cultural migrants (N = 239), and similar-culture migrants (N = 841). Most of the sample was female: 72.8% of non-migrants, 73.6% of cultural migrants, and 76.8% of similar-culture migrants were women. Concerning education, the non-migrants had received an average of 17 years of education (SD = 4.5), while the cultural migrants had received 17.7 years on average (SD = 5.0) and the similar-culture migrants 18.0 years (SD = 5.5). The majority of all three groups were retired (51.9% of non-migrants, 45.2% of cultural migrants, and 44.6% of similar-culture migrants). Most people thought that their needs were met fairly well or very well by their available financial means: 87.5% of non-migrants, 79.9% of cultural migrants, and 83.9% of similar-culture migrants.

The bivariate analyses showed significant differences in personal characteristics between the three groups. The cultural migrants (M = 62.2, SD = 5.7) were slightly younger than the non-migrants (M = 63.1, SD = 6.2) and similar-culture migrants (M = 63.6, SD = 6.7) (*p* < 0.05). The non-migrants had received the least years of education (M = 17.0, SD = 4.5) compared with the cultural migrants (M = 17.7, SD = 5.0) and similar-culture migrants (M = 18, SD = 5.5) (*p* < 0.01). The cultural migrants have the lowest income level: 20.1% rated “poorly” compared with 12.5% of non-migrants and 16.1% of similar-culture migrants (*p* < 0.05). There was no significant statistical difference between the groups in terms of gender, employment, and self-perceived health.

### 5.2. No Significant Difference in Loneliness among the Non-Migrants, Cultural Migrants, and Similar-Culture Migrants

The three groups’ loneliness levels according to the UCLA scale were not significantly different from each other. Thus, Hypothesis 1—cultural migrants have a higher loneliness level than those from a similar cultural background—was rejected. The non-migrants (N = 1084) and cultural migrants (N = 239) had the same loneliness score of 2.6 (SD = 1.2), while the similar-culture migrants (N = 841) had a loneliness score of 2.7 (SD = 1.2). This finding means that older migrants in the BBC loneliness data, regardless of their cultural origin, have similar levels of loneliness.

### 5.3. Social Capital, Discrimination, and Ageism All Significantly Associated with Loneliness across the Three Groups

Table 2 shows the results of the linear regressions for loneliness. The linear regression model of loneliness accounts for 37.3% of the variance for the group of non-migrants, 27.3% for the cultural migrants, and 29.8% for the similar-culture migrants. The effect sizes of the regression models of all the predictors on the dependent variable of loneliness for the three groups are 0.59, 0.38, and 0.42, respectively. The effect sizes are all large based on the guidelines for interpretation of Cohen’s f2, indicating that 0.02 is a small effect, 0.15 is a medium effect, and 0.35 is a large effect [40].

Social capital, discrimination, and ageism, the three elements of the social environment in our study, are all significantly associated with loneliness in older migrants regardless of their cultural background. Thus, Hypothesis 2 was rejected as all three elements of the social environment were significant predictors of loneliness across the three groups regardless of their migration background. Higher social capital appears to act as a protective factor for loneliness, because it shows a negative association for members of all three groups: non-migrants (β = −0.21, *p* < 0.001, 95% CI [−0.28, −0.12]), cultural migrants (β = −0.27, *p* < 0.005, 95% CI [−0.48, −0.05]), and similar-culture migrants (β = −0.13, *p* < 0.005, 95% CI [−0.25, −0.03]).

Experiencing discrimination is a risk factor for loneliness in older people regardless of their migration background: non-migrants (β = 0.22, *p* < 0.001, 95% CI [0.16, 0.29]), cultural migrants (β = 0.18, *p* < 0.005, 95% CI [0.02, 0.34]), and similar-culture migrants (β = 0.11, *p* < 0.005, 95% CI [0.03, 0.20]). The top four reasons that the respondents gave for the feeling of being discriminated against were age, gender, education, and ancestry and race (Table 1). There exist between-group differences in terms of the reasons for feeling discriminated against. More non-migrants felt they were being discriminated against because of age (57.5%), gender (40.8%), and education (33.6%) compared with cultural migrants (44.2%, 38.1%, and 27.5%, respectively) and similar-culture migrants (46.3%, 33.7%, and 26.4%, respectively). Significantly more cultural migrants (37.3%) gave the reason of ancestry and race for the feeling of being discriminated against compared with the non-migrants (17.2%) and similar-culture migrants (27.6%). Ageism was significantly associated with higher levels of loneliness in all three groups: non-migrants (β = 0.26, *p* < 0.001, 95% CI [0.20, 0.31]), cultural migrants (β = 0.30, *p* < 0.001, 95% CI [0.16, 0.43]), and similar-culture migrants (β = 0.30, *p* < 0.001, 95% CI [0.22, 0.36]).

### 5.4. Social Situation Acts as a Protective Factor for Loneliness in Non-Migrants and Similar-Culture Migrants

Regarding the social situation, being married or cohabitating was negatively associated with loneliness for only the non-migrants (β = −0.32, *p* < 0.001, 95% CI [−0.44, −0.20]) and similar-culture migrants (β = −0.32, *p* < 0.001, 95% CI [−0.47, −0.18]). Relational mobility showed negative correlation with the non-migrants (β = −0.18, *p* < 0.001, 95% CI [−0.27, −0.10]) and similar-culture migrants (β = −0.13, *p* < 0.005, 95% CI [−0.23, −0.10]). Neither of these two factors were significant predictors of loneliness in the cultural migrants. Thus, Hypothesis 3 was supported by the statistical results.

### 5.5. Coping Strategies: Active Coping and Non-Coping Are Significantly Associated with Loneliness

Active coping showed a negative association with loneliness in all three groups: non-migrants (β = −0.04, *p* < 0.005, 95% CI [−0.07, 0.00]), cultural migrants (β = −0.08, *p* < 0.005, 95% CI [−0.15, 0.00]), and similar-culture migrants (β = −0.04, *p* < 0.005, 95% CI [−0.08, 0.01]). Thus, adopting an active coping strategy, such as joining a club and finding new social activities, appeared protective against loneliness. The passive coping strategies showed no significant association across all three groups. Meanwhile, non-coping (i.e., not being aware of any coping strategies) was positively associated with loneliness in all three groups: non-migrants (β = 0.84, *p* < 0.001, 95% CI [0.65, 1.07]), cultural migrants (β = 0.60, *p* < 0.005, 95% CI [0.08, 1.10]), and similar-culture migrants (β = 0.75, *p* < 0.001, 95% CI [0.51, 0.99]). These findings led to the rejection of the fourth hypothesis that different coping strategies influenced differently for different groups of older people.

The top three active coping strategies adopted by the respondents in the sample were as follows: (1) joining a club (52.1% of non-migrants, 47.1% of cultural migrants, and 48.6% of similar-culture migrants); (2) deliberately starting conversations with anyone you interact with (e.g., in shops) (45.7%, 44.4%, and 48%, respectively); and (3) finding new social activities and pastimes (42.5%, 46.2%, and 43.4%, respectively). The top three passive coping strategies were: (1) dedicating time to work (63.5%, 57.3%, and 46%, respectively); (2) looking for a new job (57.3%, 58.2%, and 56.7%, respectively); and (3) changing thinking to be more positive (46.0%, 50.4%, and 44.1%, respectively). However, not all the respondents were able to engage in active or passive coping strategies. Some 9.5% of non-migrants, 8.2% of cultural migrants, and 9.6% of similar-culture migrants indicated that they were not aware of any coping strategies. There were statistically fewer similar-culture migrants (58.6%) who adopted the passive coping strategies of dedicating time to work, study, or hobbies compared with non-migrants (63.5%) and cultural migrants (64.3%) (x^2^ = 5.8; df = 2; *p* < 0.05).

### 5.6. Health, Income, and Education Show Significant Association

Three variables concerning personal characteristics were significantly associated with loneliness: self-perceived health, income, and education. Self-perceived health was negatively associated with loneliness for the non-migrants (β = −0.07, *p* < 0.001, 95% CI [−0.10, −0.05]), cultural migrants (β = −0.05, *p* < 0.005, 95% CI [−0.11, 0.00]), and similar-culture migrants (β = −0.08, *p* < 0.001, 95% CI [−0.11, −0.05]). Higher income was protective against loneliness for the cultural migrants (β = −0.42, *p* < 0.005, 95% CI [−0.79, −0.06]) and similar-culture migrants (β = −0.33, *p* < 0.005, 95% CI [−0.52, −0.12]), but not for the non-migrants. Education was negatively associated with loneliness in the group of non-migrants (β = −0.01, *p* < 0.01, 95% CI [−0.03, 0.00]), but not in the two groups of older people with a migration background.

## 6. Discussion

This study explored how culture is related to experiences of loneliness among older migrants. We focused on three groups of ageing adults (cultural migrants, similar-culture migrants, and non-migrants) and explored (1) whether the loneliness level differed among these three groups of older people and (2) what factors are associated with their experiences of loneliness. This section discusses the research findings, limitations of the study, directions for future studies, and practical interventions for loneliness.

We found no significant difference in the loneliness levels among older migrants based on the individualist-collectivist cultural indicator, as defined by Hofstede [7]. Contrary to what was expected (more cultural difference leading to higher loneliness), migrants’ culture of origin (or their cultural background) is not associated with their loneliness level. Rather, the most influential factors in terms of predicting loneliness were individual resources (as measured in terms of coping strategies), social situation (i.e., marital status and relational mobility), and the social environment of the receiving society (as measured in social capital, discrimination, and ageism).

Concerning individual resources, being aware of active coping strategies focused on initiating new social contacts is protective against loneliness across all three groups. This finding is consistent with existing literature confirming the beneficial role of active coping strategies that directly deal with the stressor at hand [41]. In terms of social status, being married or cohabitating is correlated with lower levels of loneliness, which accords with previous literature stating that marriage or cohabitation is positively associated with subjective well-being [42]. Our finding also shows that higher levels of relational mobility, or opportunities for more active engagement in close relationships, protects against loneliness. This can be explained by the fact that those engaged more actively in close relationships are also people with more social resources, and those resources can mediate the negative consequences of loneliness [43]. We also found that being childless or lacking meaningful social contact with offspring for older adults is not significantly correlated with loneliness across cultures, which is consistent with existing literature stating that childlessness does not necessarily lead to higher levels of loneliness [44,45]. The explanation for this could be that older adults have “filled the possible gap due to their childlessness with other activities or persons” [45]. These activities can be playing sports, travelling, or engaging in establishing other meaningful social networks with godchildren, nephews, nieces, or the children of friends [45].

Our study highlights the important role of the social environment for the ageing population, regardless of their migration background. This can be explained by the theory of environment gerontology, which emphasizes the person–environment relationship as decisive in ageing outcomes [46]. A favorable social environment with high social capital, where interpersonal trust and reciprocity create stronger ties among kin, friends, neighbors, and strangers alike [47], can help older adults buffer the negative experience of loneliness. The positive association of discrimination and ageism with loneliness confirms existing research findings that discrimination and ageism can result in social exclusion from support networks and be detrimental to individual well-being [22,47].

This study shows that the social environment plays a prominent role in understanding health outcomes such as loneliness. This finding merits a shift from the individual-based deficiency model to a society-based deficiency model. The determining factor for how older migrants from another culture feel in a new environment is more about the characteristics of the receiving society than about the individual migrant’s characteristics: whether they experience discrimination, whether trust between people exists, and whether they are treated negatively due to their advanced age (ageism) are what matter in predicting loneliness. Even though discrimination has no place in public opinion, until this day, discrimination toward older migrants continues to affect older migrants negatively, leading to inequality and disparity in health and social care [48]. How to address these structural barriers should be at the top of the agenda for policymakers in charge of health issues for the ageing population with a migration background. In this sense, public health policymakers can support initiatives that support an anti-discrimination and favorable social environment in the receiving society, where older migrants can thrive and achieve maximum well-being.

This overall finding challenges the unidimensional definition of loneliness only from the perspective of deficiency in social contact [3]. Our results support current efforts among social gerontological researchers [49] to broaden the loneliness research toward a multi-dimensional perspective by including structural factors, such as social environment variables, to establish a more holistic picture. Apart from being influenced by individual-level factors such as the number of social contacts, we have shown that loneliness can also be a consequence of an unfavorable social environment.

The second research objective was to explore the associations of different influencing factors with loneliness among older adults from different cultural backgrounds. We found that older migrants from different cultural backgrounds vary in their protective factors toward loneliness. This highlights the importance of categorizing older adults based on their cultural background when devising intervention programs. For instance, the social situation, as measured by marital status and relational mobility, can protect non-migrants and similar-culture migrants from loneliness, although this protective effect was not seen in the group of cultural migrants. This finding informs several suggestions for future interventions to reduce loneliness levels based on older people’s cultural background. For non-migrants and similar-culture migrants, ameliorating their social situation might be an interventional tool for decreasing their loneliness. For example, we would expect, based on our findings, that an intervention focused on increasing meaningful contact, such as having a partner, and encouraging relational mobility would work for those non-migrants and similar-culture migrants experiencing loneliness. For all older adults, regardless of their cultural background, loneliness interventions could focus on increasing knowledge of active coping strategies, such as joining a club and finding new social activities. This is in line with previous research confirming the role of new social contacts in reducing older migrants’ reports of loneliness [50].

This study is not without limitations. First, there are potential issues with the sample composition. Since the recruitment for the study was announced in English, only migrants who had no local language barrier could participate in the research. This means that older migrants who have not mastered English well enough to listen to BBC News or read English newspapers were excluded from the research. Most likely, the ones who experience language barriers are the ones who are excluded from meaningful social relationships [51] and have heightened levels of loneliness compared with those without a migration background [25]. This limitation also points to the opportunity for future studies to conduct qualitative interviews with older adults with language barriers (possibly the most socially excluded ones) to get deeper insights into their loneliness experiences. Not being able to speak the local language (i.e., having the language barrier) can lead to poor quality social relationships [51,52] and exclusion from social participation in the larger society [21]. Second, we have a rather small sample size, especially for the group of cultural migrants, in our study, which restricts the interest to test the possible differences in the predictive power of the independent variables (e.g., everyday discrimination) across the three groups. Third, the unequal sample sizes across the three groups of older people do result in reduced statistical power. However, since equal sample sizes are not a perquisite for conducting one-way ANOVA and Chi-square tests, and as our three groups have roughly the same variance [53] (pp. 375–394), we still believe in the outcome of our statistical analysis. Despite these limitations, the strength of the study also lies in BBC Loneliness Experiment, with its broad coverage of topics related to loneliness, such as the social environment, coping, and social situation, and with its respondents from various backgrounds.

## 7. Conclusions

This study broadens the research on loneliness in the ageing population through the lens of a cultural and social perspective by studying loneliness in three groups of older adults (i.e., non-migrants, cultural migrants, and similar-culture migrants) and its influencing factors. The findings show that there exists no significant difference in loneliness among the three groups. Concerning the influencing factors, a favorable social environment is protective against loneliness across the three groups, while discrimination is a risk factor. The findings point to the importance of structural factors such as the social environment in which older people find themselves rather than their culture of origin in later-life feelings of loneliness. In this sense, loneliness can be a consequence of an unfavorable social environment with low social capital and high levels of discrimination and ageism. This study calls for a shift from an individual-deficit to a society-deficit focus in scientific research into the public health problem of loneliness.

## Figures and Tables

**Table 1:** Sample descriptive information.

	Non-Migrants (NM) (N = 1084)	Cultural Migrants (CM) (N = 239)	Similar-Culture Migrants (SCM) (N = 841)	F Value or x^2^	Contrasts Tukey HSD
Variables	M (%) (SD)	M (%) (SD)	M (%) (SD)		
**Loneliness M**	2.6 (1.2)	2.6 (1.2)	2.7 (1.2)	0.3	
**Social environment**					
Social capital	3.2 (0.8)	3.2 (0.7)	3.2 (0.7)	0.8	
Discrimination	2.3 (1.0)	2.4 (0.9)	2.3 (0.9)	2.1	
Ageism	3.3 (1.1)	3.3 (1.0)	3.3 (1.0)	0.4	
**Social situation**					
Marital status (%)				5.8	
Married or cohabiting	40.2	43.0	39.8		
Divorced or separated	30.7	30.3	30.6		
Widowed	10.0	7.4	11.0		
Single	15.8	17.2	14.9		
In a relationship (not living together)	3.3	2.1	3.7		
Childless %	34.0	35.3	33.6	0.2	
Relational mobility	3.9 (0.7)	3.9 (0.7)	3.9 (0.7)	0.4	
**Coping strategies**					
Active coping	2.7 (1.9)	3.2 (2.3)	2.8 (2.2)		
Top 3 active coping strategies					
Joining a club	52.1	47.1	48.6	3.4	
Deliberately starting conversations with anyone you interact with (e.g., in shops)	45.7	44.4	48.0	1.0	
Finding new social activities and pastimes	42.5	46.2	43.4	2.7	
Passive coping	2.4 (1.9)	2.65 (2.2)	2.5 (2.1)		
Top 3 passive coping strategies					
Dedicating time to work, study, or hobbies	63.5	64.3	58.6	5.8 **	SCM ≠ NM, CM
Looking for a new job	57.3	58.2	56.7	0.2	
Change thinking to be more positive	46.0	50.4	44.1	3.0	
Non-coping	9.5	8.2	9.6	0.5	
**Personal characteristics**					
Age	63.4 (6.2)	62.2 (5.7)	63.6 (6.7)	4.4 **	CM ≠ SM, NM
Gender				4.1	
Female	72.8	73.6	76.8		
Male	27.2	26.4	23.2		
Years of education	17.0 (4.5)	17.7 (5.0)	18.0 (5.5)	11.0 ***	NM ≠ CM, SCM
Employment				16.6	
Retired	51.9	45.2	44.6		
Full-time work	22.0	26.4	26.8		
Part-time work	18.9	20.9	19.6		
Full-time or part-time student	0.6	0.4	0.7		
Non-paid work	3.4	4.2	3.8		
Unemployed	3.2	2.9	4.5		
Income				16.8 **	
Poorly	12.5	20.1	16.1		CM ≠ NM, SCM
Fairly well	47.7	50.6	48.0		CM ≠ NM, SCM
Very well	39.8	29.3	35.9		CM ≠ NM, SCM
Self-perceived health	6.4 (2.7)	6.3 (2.6)	6.1 (2.4)	2.3	

** *p* < 0.05; *** *p* < 0.01; ≠ means that differences between groups are significantly different.

**Table 2:** Linear regressions stratified by culture of origin.

	Non-Migrants (NM) (N = 1084)		Cultural Migrants (CM) (N = 239)		Similar-Culture Migrants (SCM) (N = 841)	
	B [95% CI]	SE	B [95% CI]	SE	B [95% CI]	SE
**Social Environment**						
Social Capital	−0.21 [−0.28, −0.12]	0.04 ***	−0.27 [−0.48, −0.05]	0.11 **	−0.13 [−0.25, −0.03]	0.06 **
Discrimination	0.22 [0.16, 0.29]	0.03 ***	0.18 [0.02, 0.34]	0.08 **	0.11 [0.03, 0.20]	0.04 **
Ageism	0.26 [0.20, 0.31]	0.03 ***	0.30 [0.16, 0.43]	0.70 ***	0.30 [0.22, 0.36]	0.04 ***
**Social Situation**						
Marital Status (Married or Cohabitating)	−0.32 [−0.44, −0.20]	0.06 ***	−0.20 [−0.49, 0.08]	0.14	−0.32 [−0.47, −0.18]	0.07 ***
Childness (Having a Child)	0.00 [−0.13, 0.13]	0.06	0.00 [−0.29, 0.29]	0.15	0.04 [−0.12, 0.19]	0.08
Relational Mobility	−0.18 [−0.27, −0.10]	0.05 ***	−0.09 [−0.31, 0.14]	0.12	−0.13 [−0.23, −0.10]	0.06 **
**Coping Strategies**						
Active Coping	−0.04 [−0.07, 0.00]	0.02 **	−0.08 [−0.15, 0.00]	0.04 **	−0.04 [−0.08, 0.01]	0.02 **
Passive Coping	−0.01 [−0.04, 0.03]	0.02	0.03 [−0.05, 0.10]	0.04	−0.00 [−0.05, 0.03]	0.02
Non-Coping	0.84 [0.65, 1.07]	0.11 ***	0.60 [0.08, 1.10]	0.26 **	0.75 [0.51, 0.99]	0.12 ***
**Personal Characteristics**						
Age	−0.01 [−0.02, 0.00]	0.01	0.00 [−0.03, 0.03]	0.01	−0.01 [−0.02, 0.01]	0.01
Gender	−0.06 [−0.19, 0.08]	0.07	−0.20 [−0.52, 0.12]	0.16	−0.01 [−0.16, 0.17]	0.85
Education (Years)	−0.01 [−0.03, 0.00]	0.01 *	0.01 [−0.02, 0.04]	0.01	−0.00 [−0.02, 0.01]	0.06
Employment Status (Employed)	−0.10 [−0.24, 0.04]	0.07	0.01 [−0.30, 0.32]	0.16	0.04 [−0.12, 0.22]	0.09
Income (Fairly Well or Very Well)	−0.13 [−0.31, 0.05]	0.09	−0.42 [−0.79, −0.06]	0.18 **	−0.33 [−0.52, −0.12]	0.10 **
Self-Perceived Health	−0.07 [−0.10, −0.05]	0.01 ***	−0.05 [−0.11, 0.00]	0.03 **	−0.08 [−0.11, −0.05]	0.02 ***
	Adjusted R square = 37.3%		Adjust R square = 27.3%		Adjusted R square = 29.8%	
	Cohen’s f^2^ = 0.59		Cohen’s f^2^ = 0.38		Cohen’s f^2^ = 0.42	

*** *p* < 0.001; ** *p* < 0.005; * *p* < 0.01.

## Data Availability

Data is not publicly available due to privacy restrictions.

## Appendix A. Excerpted List of Questions from BBC Loneliness Survey

**Q1. INSTRUCTIONS: Indicate how often each of the statements below is descriptive of you.**

	**Never**	**Rarely**	**Sometimes**	**Often**	**Always**
1. How often do you feel that you lack companionship?	1	2	3	4	5
2. How often do you feel left out?	1	2	3	4	5
3. How often do you feel isolated from others?	1	2	3	4	5
4. How often do you feel in tune with people around you?	1	2	3	4	5

**Q2. The following set of questions will focus on your local neighbourhood.**

**2.1 People around here are willing to help their neighbours.**

	Strongly Disagree		Strongly Agree	
	1	2	4	5
(1)				
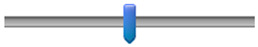

**2.2 This is a close-knit, or ‘‘tight’’ neighbourhood where people generally know one another.**

	Strongly Disagree		Strongly Agree	
	1	2	4	5
(1)				

**2.3 If I had to borrow some emergency cash, I could borrow it from a neighbour.**

	Strongly Disagree		Strongly Agree	
	1	2	4	5
(1)				

**2.4 People in this neighbourhood generally don’t get along with each other.**

	Strongly Disagree		Strongly Agree	
	1	2	4	5
(1)				

**2.5 People in this neighbourhood can be trusted.**

	Strongly Disagree		Strongly Agree	
	1	2	4	5
(1)				

**2.6 If I were sick I could count on my neighbours to shop for groceries for me.**

	Strongly Disagree		Strongly Agree	
	1	2	4	5
(1)				

**2.7 People in this neighbourhood do not share the same values.**

	Strongly Disagree		Strongly Agree	
	1	2	4	5
(1)				

**Q3. In your day-to-day life how often have any of the following things happened to you:**

**3.1 You are treated with less courtesy or respect than other people.**

	Never			Almost Every day		
	1	2	3	5	6	7
(1)						

**3.2 You receive poorer service than other people at restaurants or stores.**

	Never			Almost Every day		
	1	2	3	5	6	7
(1)						

**3.3 People act as if they think you are not smart.**

	Never			Almost Every day		
	1	2	3	5	6	7
(1)						

**3.4 People act as if they are afraid of you.**

	Never			Almost Every day		
	1	2	3	5	6	7
(1)						

**3.5 You are threatened or harassed.**

	Never			Almost Every day		
	1	2	3	5	6	7
(1)						

**Q4. What do you think is the main reason for these experiences? Please click as many as apply.**

Your ancestry or national origins (1) Your gender (2) Your race (3) Your age (4) Your religion (5) Your height (6) Your weight (7)	Physical attractiveness (8) A physical disability (9) Mental Health (10) Your sexual orientation (11) Your education or income level (12) Other (13)

**Q5. To what extent do you agree or disagree with the following statement: “Old age is a time of loneliness”.**

	Strongly Disagree		Strongly Agree	
	1	2	4	5
(1)				

**Q6. What is your marital status? Please click one.**

Single (never married or never in a civil partnership) (1)
Cohabiting (6)
In a relationship (and not living together) (11)
Married (including those in civil partnerships) (2)
Separated (but still legally married or in a civil partnership) (3)
Divorced (including formerly in a civil partnerships which is now legally dissolved) (4)
Widowed (Including surviving partner from a civil partnership) (5)

**Q7. Are you a parent?**

Yes (1)
No (2)

**Q8. For this next set of questions, we would like you to think about the people in your immediate society (your school, workplace, town, neighbourhood, etc).**

**Please indicate how true you feel each statement to be for the people around you.**

**Q8.1 They have many chances to get to know other people.**

	Strongly Disagree (1)	Moderately Disagree (2)	Slightly Disagree (3)	Slightly Agree (4)	Moderately Agree (5)	Strongly Agree (6)
(1)						
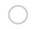

**Q8.2 It is common for these people to have a conversation with someone they have never met before.**

	Strongly Disagree (1)	Moderately Disagree (2)	Slightly Disagree (3)	Slightly Agree (4)	Moderately Agree (5)	Strongly Agree (6)
(1)						

**Q8.3 They can choose who they interact with.**

	Strongly Disagree (1)	Moderately Disagree (2)	Slightly Disagree (3)	Slightly Agree (4)	Moderately Agree (5)	Strongly Agree (6)
(1)						

**Q8.4 There are few opportunities for these people to form new friendships.**

	Strongly Disagree (1)	Moderately Disagree (2)	Slightly Disagree (3)	Slightly Agree (4)	Moderately Agree (5)	Strongly Agree (6)
(1)						

**Q8.5 It is uncommon for these people to have a conversation with people they have never met before.**

	Strongly Disagree (1)	Moderately Disagree (2)	Slightly Disagree (3)	Slightly Agree (4)	Moderately Agree (5)	Strongly Agree (6)
(1)						

**Q8.6 If they did not like their current groups, they would leave for better ones.**

	Strongly Disagree (1)	Moderately Disagree (2)	Slightly Disagree (3)	Slightly Agree (4)	Moderately Agree (5)	Strongly Agree (6)
(1)						

**Q8.7 It is often the case that they cannot freely choose who they associate with.**

	Strongly Disagree (1)	Moderately Disagree (2)	Slightly Disagree (3)	Slightly Agree (4)	Moderately Agree (5)	Strongly Agree (6)
(1)						

**Q8.8 It is easy for them to meet new people.**

	Strongly Disagree (1)	Moderately Disagree (2)	Slightly Disagree (3)	Slightly Agree (4)	Moderately Agree (5)	Strongly Agree (6)
(1)						

**Q8.9 Even if these people were not completely satisfied with the group they belonged to, they would usually stay with it anyway**.

	Strongly Disagree (1)	Moderately Disagree (2)	Slightly Disagree (3)	Slightly Agree (4)	Moderately Agree (5)	Strongly Agree (6)
(1)						

**Q8.10 These people are able to choose the groups and organisations they belong to.**

	Strongly Disagree (1)	Moderately Disagree (2)	Slightly Disagree (3)	Slightly Agree (4)	Moderately Agree (5)	Strongly Agree (6)
(1)						
